# Presenting symptoms and time to diagnosis for Pediatric Central Nervous System Tumors in Qatar: a report from Pediatric Neuro-Oncology Service in Qatar

**DOI:** 10.1007/s00381-020-04815-z

**Published:** 2020-07-24

**Authors:** Ata U. R. Maaz, Tayseer Yousif, Ayman Saleh, Ian Pople, Khalid Al-Kharazi, Jehan Al-Rayahi, Naser Elkum, Muzaffar Malik

**Affiliations:** 1 Department of Child Health, Division of Pediatric Hematology/Oncology, Sidra Medicine, Al-Luqta Street, PO Box: 26999, Doha, Qatar; 2 Department of Pediatric Surgery, Division of Neurosurgery, Sidra Medicine, Doha, Qatar; 3 Department of Radiology, Division of Neuro-imaging, Sidra Medicine, Doha, Qatar; 4 Department of Biostatistics, Sidra Medicine, Doha, Qatar; 5 Division of Medical Education, University of Brighton, Falmer, Brighton, BN1 9PH UK

**Keywords:** Child, Brain, Delay in diagnosis

## Abstract

**Introduction:**

There are no previous published reports on primary pediatric tumors of the central nervous system (CNS) in Qatar. We undertook this retrospective cohort study to review the diagnosis of CNS tumors in children in Qatar to analyze the presentation characteristics including symptoms, referral pathways, and time to diagnosis.

**Methods:**

All children registered with Pediatric Neuro-Oncology service (PNOS) were included in the study. Data from the time of diagnosis (October 2007 to February 2020) were reviewed retrospectively. Presenting symptoms were recorded and pre-diagnosis symptom interval (PSI) was calculated from the onset of the first symptom to the date of diagnostic imaging.

**Results:**

Of the 61 children registered with PNOS during the study period, 51 were included in the final analysis. Ten children were excluded because they were either diagnosed outside Qatar (*n* = 7) or were asymptomatic at the time of diagnosis (*n* = 3). The median age was 45 (range 1–171) months. Common tumor types included low-grade glioma (LGG) (47.1%) and medulloblastoma/primitive neuro-ectodermal tumors (PNET) (23.5%). Nine children had an underlying neurocutaneous syndrome. Thirty-eight patients (74.5%) had at least one previous contact with healthcare (HC) professional, but 27 (52%) were still diagnosed through the emergency department (ED). Presenting symptoms included headache, vomiting (36.2%), oculo-visual symptoms (20.6%), motor weakness (18.9%), seizures, ataxia (17.2% each), irritability, cranial nerve palsies (12% each), and endocrine symptoms (10.3%). Median PSI was 28 days (range 1–845 days) for all CNS tumors. Longest PSI was seen with germ cell tumors (median 146 days), supratentorial location (39 days), and age above 3 years (30 days). Tumor characteristics of biological behavior (high-grade tumor) and location (infratentorial) were significantly associated with shorter PSI, as were presenting symptoms of ataxia, head tilt, and altered consciousness.

**Conclusions:**

Although overall diagnostic times were acceptable, some tumor types were diagnosed after a significant delay. The awareness campaign, such as the “HeadSmart” campaign in the United Kingdom (UK), can improve diagnostic times in Qatar. Further research is required to better understand the reasons for the delay.

## Introduction

Primary CNS tumors are the largest group of solid tumors occurring in children [1–3]. They are also associated with the highest rate of cancer-related deaths in children [2, 4, 5]. The overall incidence of cancer and brain tumors in 0–14-year-old children follows the same pattern in Qatar, as elsewhere [6, 7]. Establishing the diagnosis of a CNS tumor is the crucial first step before treatment can be initiated. A delay in making a diagnosis can result in tumor progression, development of hydrocephalus, and even tentorial herniation in rare instances. If the diagnosis is delayed to the point that emergency neurosurgical intervention is required, it can lead to catastrophic consequences including less than complete surgical removal of the tumor, higher surgical morbidity and poor visual, and endocrine and neurocognitive long-term outcomes [8–11].

Diagnosing CNS tumors in children is a challenge as they are not associated with any unique diagnostic symptoms. The most common symptoms of brain tumors are headache and vomiting, which are common to many other childhood illnesses [12]. Physicians are therefore faced with the difficult decision of balancing the risk of delaying the diagnosis against that of overinvestigation and causing unnecessary alarm for the families. Identification of discriminatory symptoms can help the diagnostic process. Several international studies have examined the time to diagnosis of CNS tumors in children. The median time to diagnosis varies from 20.5 days to several months [13–22] (Table 1).

**Table 1:** Summary of recent published studies reporting diagnostic times for pediatric CNS tumors

Authors	Year published	Type of study	Population (time period)	Cohort size (age range in years)	Mean PSI	Median PSI
Reulecke et al. [13]	2008	Single center	Germany (1980–2004)	245 (0–19.2)	59.3 days	24 days
Hayashi et al. [14]	2010	Multi-center	Japan (1995–2008)	60 (1–15)	Not reported	20.5 days
Wilne et al. [15]	2012	Multi-center	UK (2004–2006)	139 (29 days–16.7 years)	Not reported	3.3 months
Shay et al. [16]	2015	Single center	Israel (1996–2004)	330 (0–18)	7.7 months	Not reported
Stocco et al. [17]	2017	Single center	Italy (2000–2012)	75 (0–16)	Not reported	4 weeks
Azizi et al. [18]	2017	Single center	Austria (2008–2013)	212 (0–19)	Not reported	60 days
Boutaher et al. [19]	2018	Single center	Morocco 2016	27 (1–15)	Not reported	2 months
Gilli et al. [20]	2019	Single center	Brazil (2005–2010)	192 (0–18)	Not reported	48 days
Patel et al. [21]	2019	Single center	USA (2008–2017)	235 (0–25)	Not reported	42 days
Hirata et al. [22]	2020	Single center	Japan (1984–2000)	85 (0–18)	Not reported	45 days

Qatar is a high-income developing country, with a population of nearly 3 million [23]. Eighty-nine percent of residents in Qatar are expatriates, with only 11% belonging to the indigenous Qatari population [24]. The proportion of Qatari children in the 0–14 year age group, however, is 32.5% [25]. The Qatar National Health Service covers the entire population through government funding and health insurance system provided by employers. The physician to population ratio and hospital beds to population ratio is among the best in the region and comparable to most industrialized nations [26]. The population is mainly urban and has easy access to primary, secondary, and tertiary healthcare including neuroimaging facilities.

According to the Qatar National Cancer Registry (QNCR) data, the incidence of brain tumors in Qatar is 15% of all childhood cancers among the 0–14-year age group [6]. Historically, children with CNS tumors in Qatar were treated at the Hamad General Hospital (HGH), which is a large teaching hospital that housed all relevant specialist services until the recent restructuring of pediatric services with the commissioning of Sidra Medicine in June 2018 as a tertiary care women and children’s hospital. Follow-up and treatment of all children with CNS tumors were transferred to Sidra Medicine and Pediatric Neuro-Oncology service (PNOS) in Qatar was also established.

There are no previous studies from Qatar reporting the presenting symptoms or diagnostic times for pediatric CNS tumors. We undertook this retrospective cohort study to review the diagnosis of CNS tumors in children in Qatar to analyze the presentation characteristics including symptoms, referral pathways, and time to diagnosis.

## Materials and methods

This is a retrospective cohort study. A review of electronic medical records (EMR) was carried out after the approval by the Institutional Review Board (IRB Number 1589712). All children up to their 18th birthday in our database who were diagnosed with a CNS tumor until the end of February 2020 were included in this study. As the majority (*n* = 29) had been diagnosed prior to the establishment of PNOS, we reviewed their medical records from the time of presentation at HGH. The earliest patient in our database was diagnosed in October 2007. The study period therefore extends between October 2007 and February 2020. Age at the time of diagnosis, gender, nationality (Qatari nationals vs. non-Qatari), and details of primary healthcare contact were also recorded. Histopathological type (where available), location of the tumor, presence of metastatic disease and presence of a neurocutaneous syndrome were noted. All presenting symptoms recorded in the medical notes and length of time (in days) each symptom had been present at the time of diagnosis was noted and analyzed if there had been symptom progression through this period. The date of diagnosis was defined as the date on which diagnostic neuroimaging was performed. Pre-diagnosis symptom interval (PSI) was defined as the time interval from the onset of the first symptom to the date of diagnosis.

### Statistical analysis

Patient characteristics are presented as numbers and percentages for each characteristic. Both mean and median are presented for the pre-diagnosis symptom interval (PSI) outcome. Because the data of PSI was highly skewed, bootstrap bias-corrected (BCa) 95% confidence intervals (CI) were computed. Student’s *t* test based on 1000 bootstrap samples was used to evaluate differences of PSI. All statistical assessments were two sided and considered significant at *p* < 0.05. All analyses were performed using Social Sciences for Windows (version 24, SPSS).

## Results

Table 2 summarizes patient characteristics. After excluding the 10 children who were either diagnosed outside Qatar (*n* = 7) or were asymptomatic at the time of diagnosis (*n* = 3), fifty-one children were included in the study. Eleven of these patients were under active therapy at the time of inclusion in this study. The median age at diagnosis was 45 months (range 1–171) with an equal gender ratio. Eighteen (35.3%) patients belonged to the native Qatari population, while the remaining 33 (64.7%) were non-Qataris. Nine patients (17.6%) had neurocutaneous syndromes (6 had tuberous sclerosis (TS) and 3 had neurofibromatosis type 1 (NF)).

**Table 2:** Patient characteristics

Characteristic	Number (%)
Total Patients	51 (100)
Median age (range)	45 months (1–171)
Up to 3 years	20 (39.2)
3 to 10 years	22 (43.1)
Above 10 years	9 (17.6)
Gender	
Male	26 (50.9)
Female	25 (49.1)
Nationality	
Qatari Nationals	18 (35.3)
Non-Qatari Nationals	33 (64.7)
Neurocutaneous syndromes	
Neurofibromatosis type 1	3 (5.6)
Tuberous sclerosis	6 (11.8)
None	42 (82.4)
Tumor location	
Supratentorial	30 (58.8)
Infratentorial	17 (33.3)
Spinal	4 (7.8)
Tumor type	
LGG	24 (47.05)
Medulloblastoma/PNET	12 (23.5)
Germ cell tumor	5(9.8)
HGG	4 (7.8)
CNS ATRT	2 (3.9)
Others	4 (7.8)
Localized/metastatic	
Localized	42 (82.4)
Metastatic/multifocal	9 (17.6)
Intracranial pressure (ICP)	
Normal	28 (54.9)
Raised	19 (37.3)
Data not available	4 (7.8)
ED vs. elective diagnosis	
ED	27 (52.9)
Elective	24 (47.1)

*LGG*, low-grade glioma; *PNET*, primitive neuro-ectodermal tumor; *HGG*, high-grade glioma; *ATRT*, atypical teratoid rhabdoid tumor; *ED*, emergency department; others (*n* = 4): choroid plexus carcinoma, 1; craniopharyngioma, 1; ependymoma, 1; and meningioma, 1

### Tumor characteristics

Diagnosis for 37 patients (72.5%) was confirmed with histopathology, whereas the remaining 14 (27.5%) patients were diagnosed on neuroimaging alone (5 each with subependymal giant cell astrocytoma (SEGA) and with optic pathway glioma (OPG), 2 with (DIPG) and 2 with other LGG. The most common tumor type in our cohort was LGG (47%), followed by medulloblastoma/primitive neuro-ectodermal tumor (PNET) (23.5%), intracranial germ cell tumor (GCT) (9.8%), high-grade glioma (HGG) (7.8%), and atypical teratoid rhabdoid tumor (ATRT) (3.9%). Thirty (58.8%) tumors were in supratentorial locations, 17 (33.3%) were infratentorial, and 4 (7.8%) in the spinal cord. Forty-two (82.4%) patients had localized tumors at the time of presentation. The remaining 9 patients had multifocal (*n* = 7) or metastatic (*n* = 2) disease. The two truly metastatic tumors included one case each of medulloblastoma and GCT, whereas the 7 multifocal tumors included 4 SEGA and 3 optic pathway glioma (OPG).

### Referral pathways

Table 3 summarizes referral pathways for the diagnosis of CNS tumors for our cohort. Thirty-eight (74.5%) had at least one prior healthcare contact before the diagnostic imaging was initiated. Documented contacts included with Pediatric Neurology (18), General Pediatrics (8), Primary Care Health Centers (8), Pediatric Endocrinology (5), Pediatric Emergency Centers (5), Ophthalmology/Opticians (4), Gastroenterology (1), Cardiology (1), and Neurosurgery (1). Twenty-eight patients were seen by a single physician/team, 5 were seen by 2 teams, and 4 patients were seen by three different teams during the symptom interval. Fourteen of the patients with prior healthcare contact were eventually diagnosed through ED, with an equal distribution of high- and low-grade tumors (7 each). The remaining 13 (25.4%) patients presented acutely to the ED without any prior healthcare contact. Nine (62%) of these had high-grade tumors (6 medulloblastoma, 2 DIPG, and 1 ATRT).

**Table 3:** Referral pathways

	Elective referrals (24)	Diagnosed through ED (27)	
Prior HC contact	Yes (24)	Yes (14)	No (13)
Median PSI (days)	33.5	19.0	
		39.5	14.0
Tumor grade			
High grade (%)	3 (12.5)	7 (50)	9 (69.2)
Low grade (%)	21 (87.5)	7 (50)	4 (30.8)
Breakdown of prior HC contact	Neuro: 12 Gen Peds, Neuro, Ophth: 2 PCHC: 3 Gen Peds, Endo: 1 Gen Peds, Neuro, Gastro: 1 Gen Peds, Neuro, Endo: 1 Gen Peds, Ophth: 1 Neuro, Cardio: 1 Ophth: 1 NS: 1	PCHC: 5 PEC: 5 Gen Peds, Endo: 2 Endo: 1 Neuro: 1	

*HC*, healthcare; *ED*, emergency department; *PSI*, pre-diagnosis symptom interval; *Neuro*, pediatric neurology; *Gen Peds*, general pediatrics; *Ophth*, pediatric ophthalmology; *PCHC*, primary care health center; *Endo*, pediatric endocrinology; *Gastro*, pediatric gastroenterology; *Cardio*, pediatric cardiology; *NS*, neurosurgery; *PEC*, primary emergency center

### Presenting symptoms

**Fig. 1:**
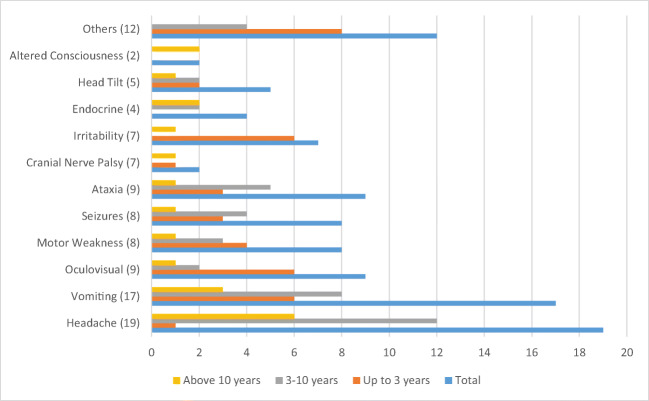
Frequency of presenting symptoms according to age (*N* = 51). Oculo-visual symptoms: nystagmus, 3; poor vision, 3; diplopia, 1; blurred vision, 1; proptosis, 1; endocrine symptoms: polyuria, 1; polydypsia, 1; precocious puberty, 1; short stature, 1. Others: back pain, 1; lethargy, 3; rash, 2; neck pain, 2; weight loss, 2; poor oral intake, 1; fever, 1

**Fig. 2:**
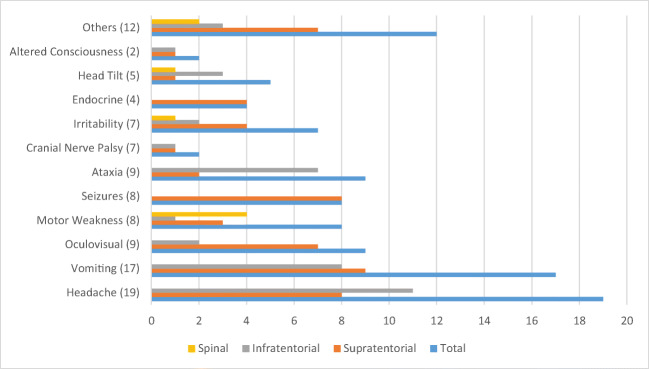
Frequency of presenting symptoms according to tumor location (*N* = 51). Oculo-visual symptoms: nystagmus, 3; poor vision, 3; diplopia, 1; blurred vision, 1; proptosis, 1; endocrine symptoms: polyuria, 1; polydypsia, 1; precocious puberty, 1; short stature, 1. Others: back pain, 1; lethargy, 3; rash, 2; neck pain, 2; weight loss, 2; poor oral intake, 1; fever, 1

Figures 1 and 2 summarize the presenting symptoms in our cohort. The median number of recorded symptoms was 2 (range 1–4). All patients had at least 1 symptom at the time of diagnosis, 39 (76.4%) had two, 19 (37.2%) had three, while 7 (13.7%) patients had developed four symptoms by the time the diagnosis was made.

The three most common presenting symptoms were headache (37.2%), vomiting (33.3%), and ataxia and oculo-visual symptoms (17.6% each). For children under 3 years of age, the most common presenting symptom was irritability, vomiting, and oculo-visual symptoms (27.2%). Headache (62%), vomiting (37.9%), and ataxia (20.6%) were the predominant presenting symptoms for older children. Figure 2 shows the frequency of symptoms according to tumor location. Supratentorial tumors presented most commonly with vomiting (30%), headache, and seizures (26.6% each), whereas the most common presenting symptoms for infratentorial tumors were headache (64.7%), vomiting (47%), and ataxia (41.1%). All spinal tumors presented with motor weakness (*n* = 4), in addition to head tilt (1) and back pain (1).

### Symptom progression

**Fig. 3:**
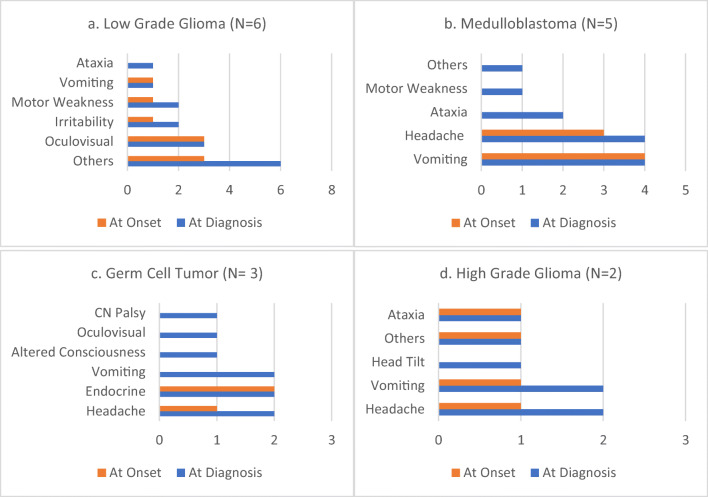
Symptom progression for common tumor types (*N* = 19). Bar charts for 3 other patients (1 each with atypical teratoid/rhabdoid tumor (ATRT), choroid plexus carcinoma, and meningioma) not shown

Symptom progression was seen in 19 (37.2%) patients. LGG was the commonest tumor type, followed by medulloblastoma, GCT, and DIPG. Three patients had other tumor types. Median PSI was 36 days for patients who had symptom progression. Medulloblastoma and ATRT were associated with the shortest PSI, whereas GCT and LGG had the longest PSI. At symptom onset, the two most common symptoms were headache (26%) and vomiting (21%). LGG had varied symptoms at onset as well as a diagnosis. Four of the five patients with medulloblastoma had vomiting and 3 had headaches at symptom onset. By the time the diagnosis was established, a fourth patient had developed a headache, two had developed ataxia, and two had developed other symptoms. Figure 3 summarizes the pattern of symptom progression in the four most common tumor types.

### Pre-diagnosis symptom interval

**Fig. 4:**
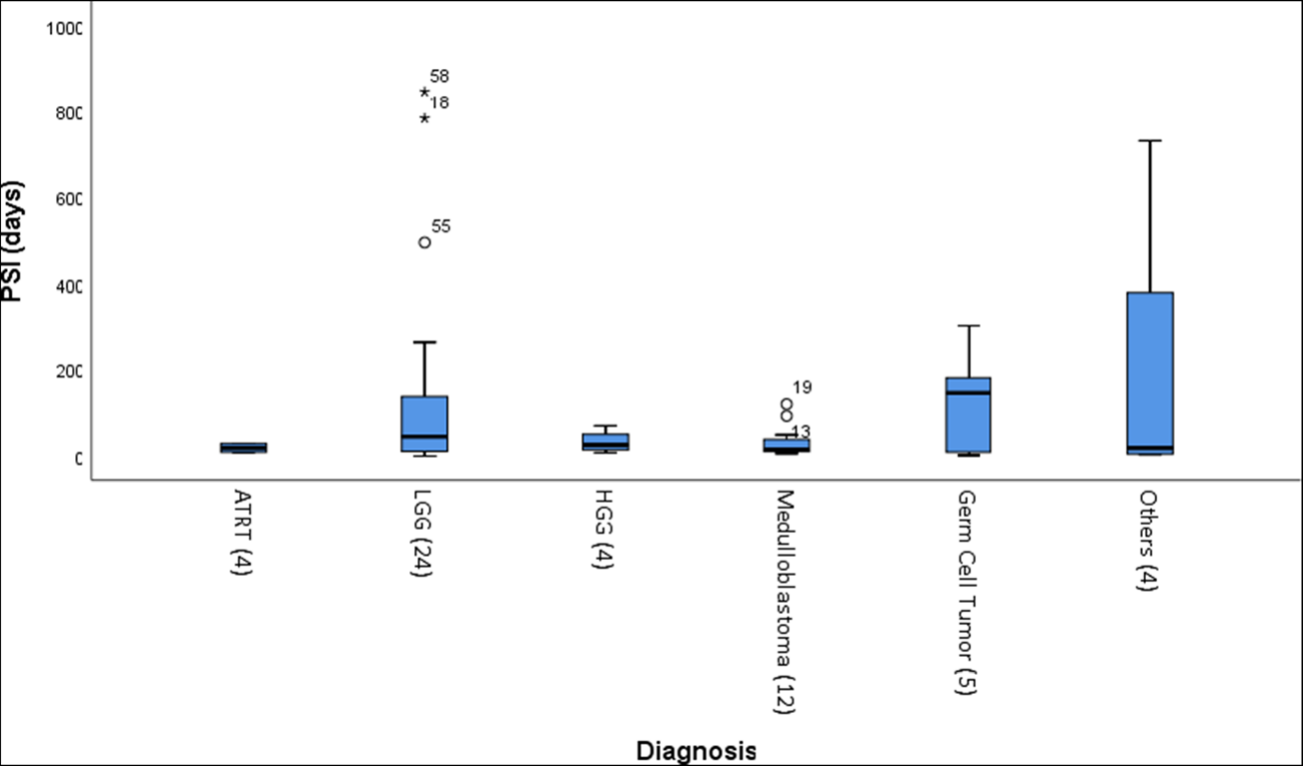
Boxplot of pre-diagnosis symptom interval according to the tumor type (*N* = 51). Median pre-diagnostic symptom intervals (PSI) according to tumor type: atypical teratoid/rhabdoid tumor (ATRT); low-grade glioma (LGG); high-grade glioma (HGG); medulloblastoma/primitive neuro-ectodermal tumor (PNET); intracranial germ cell tumor (GCT)

Figure 4 and Tables 4 and 5 summarize the PSI analysis. Median PSI was 28 days (range 1 to 845 days). Longest PSI (median 146 days) was seen with GCT (median 146 days) followed by LGG (median 45 days), HGG (median 25.5 days), and medulloblastoma/PNET (median 15 days). Age below 3 years had shorter PSI (36 days vs 13.5 days for younger age group). Infratentorial tumor location (median 39.5 days vs. 15 days for infratentorial location, *p* = 0.049) and high-grade tumor (45 days vs 15 days for high-grade tumor, *p* = 0.040) were significantly associated with a shorter PSI.

**Table 4:** Pre-diagnosis symptom interval (PSI) by patient and tumor variables (*N* = 51)

Variable	No. of cases (%)	Mean (95% CI^1^)	Median days	*p* value^a^
Age				
Up to 3 years	22 (43.14)	85.50 (24.25–156.04)	13.50	0.594
Above 3 years	29 (56.86)	117.83 (58.34–188.68)	36.00	
Sex				
Male	26 (50.98)	98.19 (52.55–157.06)	26.50	0.838
Female	25 (49.02)	109.80 (31.70–189.86)	29.00	
Nationality				
Qatari Nationals	18 (35.29)	84.39 (27.48–176.35)	22.50	0.589
Non-Qatari Nationals	33 (64.71)	114.52 (57.17–183.70)	29.00	
ED vs. elective diagnosis				
ED	27 (52.94)	54.78 (30.75–83.52)	19.00	0.113
Elective	24 (47.06)	159.13 (62.18–278.60)	33.50	
Tumor location				
Supratentorial	30 (58.82)	148.07 (70.78–244.70)	39.50	*0.049*
Infratentorial	21 (41.18)	40.76 (19.21–69.74)	15.00	
Tumor grade				
Low-grade tumor	32 (62.75)	148.03 (79.57–223.46)	45.00	*0.040*
High-grade tumor	19 (37.25)	29.53 (16.76–43.00)	15.00	
Localized vs. metastatic				
Localized	42 (82.35)	82.29 (40.24–140.91)	26.50	0.230
Metastatic	9 (17.65)	204.67 (53.14–396.00)	90.00	
Intracranial pressure (ICP)^2^				
Raised	28 (59.57)	56.89 (33.47–85.04)	24.00	0.077
Normal	19 (40.43)	191.21 (82.40–331.04)	51.00	

^1^Bootstrap bias-corrected (BCa) 95% CI based on 1000 bootstrap samples

^2^Data was available for 47 patients

^a^Independent sample *t* test based on 1000 bootstrap samples (significant values in italics)

**Table 5:** Pre-diagnosis symptom interval (PSI) by presenting symptoms (*N* = 51)

Symptom	No. of cases (%)	Mean days (95% CI^1^)	Median days (days)	*p* value^a^
Headache	19 (37.25)	93.68 (35.86–165.56)	19.00	0.793
Vomiting	17 (33.33)	82.00 (18.01–169.64)	14.00	0.585
Irritability	7 (13.73)	124.86 (3.42–394.55)	8.00	0.842
Oculo-visual	9 (17.65)	204.00 (26.34–417.87)	36.00	0.282
Ataxia	9 (17.65)	30.78 (11.70–53.58)	19.00	*0.039*
Developmental delay	2 (3.92)	455.50 (260.25–650.75)	455.50	0.280
Endocrine	4 (7.84)	339.75 (160.00–584.75)	241.00	0.080
Seizure(s)	8 (15.69)	137.13 (23.02–316.32)	39.00	0.732
Motor weakness	8 (15.69)	62.38 (17.38–129.04)	29.50	0.276
Head tilt	5 (9.80)	7.80 (3.81–11.50)	8.00	*0.021*
Cranial nerve palsy	2 (3.92)	97.00 (65.00–139.00)	97.00	0.942
Altered consciousness	2 (3.92)	7.00 (6.00–8.00)	7.00	*0.018*
General symptoms	12 (23.53)	86.75 (18.80–175.55)	14.50	0.703

^1^Bootstrap bias-corrected (BCa) 95% CI based on 1000 bootstrap samples

^a^Independent sample *t* test based on 1000 bootstrap samples (significant values in italics)

For presenting symptoms, developmental delay (*n* = 2, median 455.5 days) was associated with the longest PSI, followed by endocrine symptoms (median 241 days), seizures (47 days), and oculo-visual symptoms (43 days). Irritability (8.0 days), vomiting (14.0 days), and headache (19 days) had the shortest PSI. Raised ICP in our cohort was not associated with shorter PSI. Ataxia (*p* = 0.039), head tilt (*p* = 0.021), and altered consciousness (*p* = 0.018) were significantly associated with shorter PSI.

PSI according to the referral pathway was shorter for those who were diagnosed through ED (19.0 days vs. 33.5 days for those who were diagnosed through non-elective pathways). This difference was not statistically significant (*p* = 0.113).

## Discussion

This is the first report on primary CNS tumors in children from Qatar. We have documented the presenting symptoms and diagnostic times and highlighted some areas for future research.

Presenting symptoms of CNS tumors depend on the patient’s age, tumor location, and its biological behavior. Timely recognition of these symptoms can initiate the referral process and avoid the anxiety and uncertainty of a lengthy diagnostic process for the patient/family, and save the health service precious time, personnel, and financial resources. Our results showed symptoms of raised intracranial pressure including headache, vomiting, lethargy, and visual disturbance were common to children of all ages and all intracranial tumors. In older children, headache and vomiting were the two most commonly reported symptoms, followed by abnormal gait and oculo-visual symptoms. In children younger than 3 years of age vomiting, irritability, ocular abnormalities, and nonspecific symptoms including lethargy were more commonly seen. Infratentorial tumors were more commonly associated with symptoms of raised intracranial pressure while seizures were more common in supratentorial tumors. The frequency of presenting symptoms in our cohort is similar to most other published reports [27, 28]. Not surprisingly, the 4 children with spinal tumors in our cohort presented with symptoms of motor weakness, back pain, and head tilt.

Median PSI of 28 days in our cohort compares favorably with other published series (Table 5). Our results confirm that known factors including presenting symptoms, location, and grade of the tumor significantly influence PSI. Vomiting and seizures were associated with short PSI, whereas endocrine symptoms were associated with longer PSI. Rapidly growing tumors and age group younger than three years were associated with a shorter PSI. In our cohort, patients with the metastatic and multifocal disease were grouped together. Of the two metastatic patients in this group, one with medulloblastoma had a short PSI (15 days), while the other with GCT had a long PSI (181 days). These results are consistent with previous reports of short PSI with higher stage medulloblastoma by Halperin et al. [29, 30] and a long PSI associated with metastatic GCT by Sethi et al. [31].

Previous studies have shown long PSI to result from delay in referral for neuroimaging by physicians and to lack of access to neuroimaging facilities, which may be secondary to geographical, financial, or health service-related constraints. Closeness to a tertiary center and ready access to neuroimaging have previously been cited as determinants of early diagnosis [13, 14]. We believe that small population size, high physician to population ratio, urban habitation, and easy access to neuroimaging are among the factors leading to short PSI in our cohort.

### Importance of early diagnosis

While there is a consensus that CNS tumors should be diagnosed without delay, an “ideal” or acceptable time for diagnosis of pediatric CNS tumors is far from clear. The presumption that “early” diagnosis should lead to favorable outcomes is not necessarily true, as tumor outcomes are increasingly associated with tumor biology and metastatic stage. Biologically aggressive tumors tend to present early without being associated with improved survival. In fact, some studies report a correlation of shorter PSI with poor survival [29, 30, 32, 33]. Nonetheless, the importance of early diagnosis can also not be underestimated. A long PSI leads to symptom progression, increased intracranial pressure, and development of other specific symptoms related to the involved area of the brain. End organ damage such as visual loss, hypopituitarism, and permanent motor function or cognitive function loss are some of the other catastrophic sequelae of late diagnosis of a brain tumor [8, 10]. Early recognition of presenting signs and symptoms would lead to a timely diagnosis.

Some 20–30% of children with CNS tumors are diagnosed after presenting acutely to the ED without any prior healthcare contact due to their rapid rate of progression [15]. Our results confirm this pattern of presentation. Additionally, 14 (27%) of our patients were diagnosed through ED despite prior HC contact. Patients presenting directly to ED had a shorter PSI (14 days vs. 39 days) and a larger proportion of high-grade tumors (62% vs. 50%) compared those who had prior healthcare contact. Possible explanations for children with prior HC contact presenting to ED include lack of early recognition of presenting symptoms by the HC professionals and a sudden deterioration in the clinical condition of the child. Multiple visits to healthcare providers with the same or progressive symptoms are known to cause a perception of delay that leads to undermining of trust in the healthcare professionals [34, 35].

These findings highlight the need for increased awareness among the general public, primary care physicians, and other healthcare professionals for signs and symptoms of CNS tumors. Researchers in the United Kingdom found diagnostic time for pediatric CNS tumors to be unacceptably long at 14.4 weeks in 2006. After a comprehensive investigative and consultative process, evidence-accredited guidelines for clinicians were published in 2008 [36]. A national campaign “HeadSmart: Be brain tumour aware” was launched in 2011, which has resulted in a significant reduction of diagnostic time from 14.4 weeks in 2006 to 6.7 weeks in 2013 [17, 37, 38]. A locally adapted similar campaign to educate parents and healthcare professionals in all settings will be helpful in Qatar to recognize the signs and symptoms of CNS tumors and optimize diagnostic times. A high index of suspicion is required in all settings for recognition of the potential of a brain tumor as a possible diagnosis in children to avoid unnecessary delay in diagnosis.

## Conclusions

In Qatar, the diagnostic times for pediatric CNS tumors are short and comparable to other recent reports from Japan, Germany, and Austria. However, many children are still diagnosed with a significant delay particularly those presenting with endocrine symptoms and slow-growing tumors. Children with low-grade tumors may not be at risk of death from a delay in diagnosis but can have devastating quality of life (QoL) consequences including severe visual impairment and avoidable lifelong need for hormone replacement. Targeted awareness campaign such as *HeadSmart* can help parents and physicians recognize signs and symptoms of CNS tumors and reduce diagnostic times.

### What this study adds

This is the first study of its kind from the state of Qatar and provides a baseline for future research. In analyzing the diagnostic times, we have identified the groups of patients who are at risk of diagnostic delay and long-term morbidity. Although not a primary objective of this study, we have also been able to document the pattern of pediatric CNS tumor presentation in Qatar. Our results can be used in future epidemiological research.

### Limitations

Our data were subject to parental recall bias as defining the exact symptom onset varies depending on parental health literacy, symptoms awareness, health-seeking behavior, family support networks, and parental anxiety. In common with all retrospective studies using medical notes, we were limited by the quality of data recorded at the time of presentation. We were unable to document the symptom progression in depth due to the unavailability of pre-hospital healthcare records. Details of primary care or outpatient visits before being referred for neuroimaging were missing, making it difficult in some cases to determine the referral pathways and examine if there was a delay at the physician or healthcare level. Some data on original neuroimaging was unavailable making the ICP data incomplete. These deficiencies will be addressed in a future prospectively designed study with a larger cohort.

## References

[1] Siegel RL, Miller KD, Jemal A (2018). Cancer statistics, 2018. CA Cancer J Clin.

[2] Cancer Research UK https://www.cancerresearchuk.org/health-professional/cancer-statistics/childrens-cancers#heading-One Accessed April 2020

[3] Ostrom QT, Gittleman H, Truitt G, Boscia A, Kruchko C, Barnholtz-Sloan JS (2018). CBTRUS statistical report: primary brain and other central nervous system tumors diagnosed in the United States in 2011-2015. Neuro Oncol.

[4] Johnson KJ, Cullen J, Barnholtz-Sloan JS, Ostrom QT, Langer CE, Turner MC, McKean-Cowdin R, Fisher JL, Lupo PJ, Partap S, Schwartzbaum JA, Scheurer ME (2014). Childhood brain tumor epidemiology: a brain tumor epidemiology consortium review. Cancer Epidemiol Biomarkers Prev.

[5] Ghizoni E, Naccarato CRM, Mathias RN, Joaquim AF, Ghizoni E, Tedeschi H, Ferreira MAT (2019). Brain tumors in children. Fundamentals of neurosurgery: a guide for clinicians and medical students.

[6] 2014 Cancer Incidence Report, State of Qatar. In: National Cancer Program QnCR (ed). Ministry of Public Health, Qatar, Doha, State of Qatar, pp 86-87

[7] Belgaumi AF, Pathan GQ, Siddiqui K, Ali AA, Al-Fawaz I, Al-Sweedan S, Ayas M, Al-Kofide AA (2019). Incidence, clinical distribution, and patient characteristics of childhood cancer in Saudi Arabia: a population-based analysis. Pediatr Blood Cancer.

[8] Yule SM, Hide TA, Cranney M, Simpson E, Barrett A (2001). Low grade astrocytomas in the West of Scotland 1987-96: treatment, outcome, and cognitive functioning. Arch Dis Child.

[9] Chou SY, Digre KB (1999). Neuro-ophthalmic complications of raised intracranial pressure, hydrocephalus, and shunt malfunction. Neurosurg Clin N Am.

[10] Armstrong GT (2010). Long-term survivors of childhood central nervous system malignancies: the experience of the Childhood Cancer Survivor Study. Eur J Paediatr Neurol.

[11] Lassaletta A, Bouffet E, Mabbott D, Kulkarni AV (2015). Functional and neuropsychological late outcomes in posterior fossa tumors in children. Childs Nerv Syst.

[12] Wilne SH, Ferris RC, Nathwani A, Kennedy CR (2006). The presenting features of brain tumours: a review of 200 cases. Arch Dis Child.

[13] Reulecke BC, Erker CG, Fiedler BJ, Niederstadt TU, Kurlemann G (2008). Brain tumors in children: initial symptoms and their influence on the time span between symptom onset and diagnosis. J Child Neurol.

[14] Hayashi N, Kidokoro H, Miyajima Y, Fukazawa T, Natsume J, Kubota T, Kojima S (2010). How do the clinical features of brain tumours in childhood progress before diagnosis?. Brain Dev.

[15] Wilne S, Collier J, Kennedy C, Jenkins A, Grout J, Mackie S, Koller K, Grundy R, Walker D (2012). Progression from first symptom to diagnosis in childhood brain tumours. Eur J Pediatr.

[16] Shay V, Fattal-Valevski A, Beni-Adani L, Constantini S (2012). Diagnostic delay of pediatric brain tumors in Israel: a retrospective risk factor analysis. Childs Nerv Syst.

[17] Stocco C, Pilotto C, Passone E, Nocerino A, Tosolini R, Pusiol A, Cogo P (2017). Presentation and symptom interval in children with central nervous system tumors. A single-center experience. Childs Nerv Syst.

[18] Azizi AA, Heßler K, Leiss U, Grylli C, Chocholous M, Peyrl A, Gojo J, Slavc I (2017). From symptom to diagnosis-the prediagnostic symptomatic interval of pediatric central nervous system tumors in Austria. Pediatr Neurol.

[19] Boutahar FZ, Benmiloud S, El Kababri M, Kili A, El Khorassani M, Allali N, Khattab M, Qaddoumi I, Hessissen L (2018). Time to diagnosis of pediatric brain tumors: a report from the Pediatric Hematology and Oncology Center in Rabat, Morocco. Childs Nerv Syst.

[20] Gilli IO, Joaquim AF, Tedeschi H, Dos Santos AS, Morcillo AM, Ghizoni E (2019). Factors affecting diagnosis of primary pediatric central nervous system neoplasias in a developing country. Childs Nerv Syst.

[21] Patel V, McNinch NL, Rush S (2019). Diagnostic delay and morbidity of central nervous system tumors in children and young adults: a pediatric hospital experience. J Neurooncol.

[22] Hirata K, Muroi A, Tsurubuchi T, Fukushima H, Suzuki R, Yamaki Y, Ishikawa E, Matsumura A (2020) Time to diagnosis and clinical characteristics in pediatric brain tumor patients. Child Nerv Syst. 10.1007/s00381-020-04573-y 10.1007/s00381-020-04573-y 32157367

[23] Planning and Statistics authority (2019) Population and social statistics. https://www.psa.gov.qa/en/statistics/Statistical%20Releases/Population/Population/2019/Population_social_1_2019_AE.pdf. Date Accessed May 2019

[24] Planning and Statistics authority (2020) Qatar monthly statistics. https://www.psa.gov.qa/en/Pages/default.aspx. Date Accessed May 2020

[25] Gulf Labour Markets and Migration Qatar (2015) Population by Nationality (Qatari/non-Qatari and five-year age group (2015). Gulf Research Center. https://gulfmigration.org/qatar-population-nationality-qatari-non-qatari-five-year-age-group-2015/. Date Accessed June 2015

[26] World Health Organization (2020) Global Health Observatory Data repository/medical doctors. https://apps.who.int/gho/data/node.main.HWFGRP_0020?lang = en. Date Accessed May 2020

[27] Wilne S, Collier J, Kennedy C, Koller K, Grundy R, Walker D (2007). Presentation of childhood CNS tumours: a systematic review and meta-analysis. Lancet Oncol.

[28] Klitbo DM, Nielsen R, Illum NO, Wehner PS, Carlsen N (2011). Symptoms and time to diagnosis in children with brain tumours. Dan Med Bull.

[29] Halperin EC, Friedman HS (1996). Is there a correlation between duration of presenting symptoms and stage of medulloblastoma at the time of diagnosis?. Cancer.

[30] Halperin EC, Watson DM, George SL (2001). Duration of symptoms prior to diagnosis is related inversely to presenting disease stage in children with medulloblastoma. Cancer.

[31] Sethi RV, Marino R, Niemierko A, Tarbell NJ, Yock TI, MacDonald SM (2013). Delayed diagnosis in children with intracranial germ cell tumors. J Pediatr.

[32] Kukal K, Dobrovoljac M, Boltshauser E, Ammann RA, Grotzer MA (2009). Does diagnostic delay result in decreased survival in paediatric brain tumours?. Eur J Pediatr.

[33] Freeman CR, Krischer JP, Sanford RA, Cohen ME, Burger PC, del Carpio R, Halperin EC, Munoz L, Friedman HS, Kun LE (1993). Final results of a study of escalating doses of hyperfractionated radiotherapy in brain stem tumors in children: a Pediatric Oncology Group study. Int J Radiat Oncol Biol Phys.

[34] Dixon-Woods M, Findlay M, Young B, Cox H, Heney D (2001). Parents’ accounts of obtaining a diagnosis of childhood cancer. Lancet.

[35] Mazor KM, Roblin DW, Greene SM, Lemay CA, Firneno CL, Calvi J, Prouty CD, Horner K, Gallagher TH (2012). Toward patient-centered cancer care: patient perceptions of problematic events, impact, and response. J Clin Oncol.

[36] Wilne S, Koller K, Collier J, Kennedy C, Grundy R, Walker D (2010). The diagnosis of brain tumours in children: a guideline to assist healthcare professionals in the assessment of children who may have a brain tumour. Arch Dis Child.

[37] Shanmugavadivel S, Walker D, Liu J-F, Wilne S (2015) HeadSmart: are you brain tumour aware? Paediatr Child Health 26:81–86

[38] Walker D, Wilne S, Grundy R, Kennedy C, Neil DA, Lindsell S, Trusler J, Evans A, Dudley J, Thomson A, Lakhanpaul M, Clough L, Baker M, Chu T, Liu J-F, Pearson E, Rayner E, Thorne E, Franklin S (2015). A new clinical guideline from the Royal College of Paediatrics and Child Health with a national awareness campaign accelerates brain tumor diagnosis in UK children—“HeadSmart: be brain tumour aware”. Neuro-Oncology.

